# Serum calcium levels are associated with cognitive function in hypoparathyroidism: a neuropsychological and biochemical study in an Italian cohort of patients with chronic post-surgical hypoparathyroidism

**DOI:** 10.1007/s40618-022-01822-6

**Published:** 2022-06-25

**Authors:** F. Saponaro, G. Alfi, F. Cetani, A. Matrone, L. Mazoni, M. Apicella, E. Pardi, S. Borsari, M. Laurino, E. Lai, A. Gemignani, C. Marcocci

**Affiliations:** 1 Department of Surgical, Medical, and Molecular Pathology and Critical Care Medicine, University of Pisa, Pisa, Italy; 2 University Hospital of Pisa, Endocrine Unit 2, Pisa, Italy; 3 Department of Clinical and Experimental Medicine, University of Pisa, Pisa, Italy

**Keywords:** Neurocognitive evaluation, Post-surgical hypoparathyroidism, Hypoparathyroidism, Parathyroid hormone

## Abstract

**Purpose:**

Hypoparathyroidism (HypoPT) is a rare endocrine disease and conventional therapy is based on calcium and vitamin D analogues. Conventional therapy does not restore calcium homeostasis and patients complain with neuropsychological symptoms, which have been evaluated with nonspecific self-administered questionnaires. This study aims to evaluate cognitive functions of patients with chronic post-surgical (PS)-HypoPT compared to a control population, using a standardized neuropsychological approach and evaluating the relationship with serum calcium (Alb-Ca).

**Methods:**

Observational, monocentric study on 33 patients with PS-HypoPT and 24 controls, in whom biochemical testing and a standardized neuropsychological assessment by a trained psychologist were performed.

**Results:**

In patients with PS-HypoPT, low Alb-Ca correlated with a worse performance on semantic memory abilities and executive function, as suggested by a significant inverse correlation between Alb-Ca and Trail Making Test A (TMT-A) scores (*r* = − 0.423; *p* = 0.014) and by a positive correlation with Semantic Fluency Test scores (SF)(*r* = 0.510; *p* = 0.002). PS-HypoPT patients with Alb-Ca ≤ 8.9 mg/dl had a significantly lower test performance compared with PS-HypoPT patients with Alb-Ca > 8.9 mg/dl, both at the TMT-A test (mean score: 34.53–18.55; *p* < 0.0001) and at SF test (mean score: 41.94–48.68; *p* = 0.01) and also a significantly lower test performance compared with control patients’ group at TMT-A (mean score: 34.53–25.5; *p* = 0.0057).

**Conclusions:**

Patients with chronic PS-HypoPT in conventional therapy do not show a severe cognitive impairment; however, cognitive functions namely visuo-spatial attention, executive function and semantic memory appear to be modulated by Alb-Ca and impaired by its low levels.

## Introduction

Hypoparathyroidism (HypoPT) is a rare endocrine disease which is characterized by hypocalcaemia and undetectable or inappropriately low serum parathyroid hormone (PTH) [1]. Post-surgical HypoPT (PS-HypoPT) is the most common cause of HypoPT and is caused by accidental parathyroid removal/injury during neck surgery [2]. PTH displays a crucial role in the maintenance of calcium homeostasis through the effects on kidney reabsorption of calcium and phosphate excretion, on skeletal regulation of bone turnover and indirect effect on gastrointestinal calcium absorption, vitamin D mediated [1, 3].

The lack of PTH in patients with HypoPT profoundly changes calcium homeostasis and patients are at risk of hypocalcaemia, hypercalciuria, hyperphosphatemia and abnormal skeletal mineralization [4]. This results in chronic clinical manifestations of multiple organs, mainly renal complications (nephrolithiasis and decline of kidney function), reduced bone turnover and bone quality, increased risk of cardiovascular disease, neuromuscular impairment and neuropsychological symptoms with poor quality of life [5–7].

Conventional therapy, based on calcium supplements and activated vitamin D, does not completely restore calcium homeostasis [8]. A step forward has been made with the approval of recombinant human PTH (1–84) [rhPTH(1–84)] for those patients who are not controlled on conventional therapy.

From a neuropsychological standpoint, patients with HypoPT complain with cognitive and affective symptoms such as brain fog, impaired ability to focus, memory loss, depression, anxiety and fatigue [9, 10]. As well described and hypothesized in the literature, the more plausible pathophysiological mechanism resides in a direct effect of PTH in the central nervous system. PTH can modulate calcium flux, crucial for the correct functioning of the brain cells, and also phosphate homeostasis, by sodium dependent phosphate transporter 2 (Pi2). It has been also described that PTH could influence regional cerebral capillary blood flow, whose changes in HypoPT could explain alterations in neurovascular coupling [11]. Finally, there is also some evidence that PTH crosses the blood brain barrier interacting with neurons expressing PTH receptors (particularly PTH receptor2—PTHR2) [12, 13].

Cognitive symptoms in patients with HypoPT have been evaluated as part of quality of life (QoL) assessment. In several studies, patients with Hypo-PT showed a consistent reduction of QoL compared to both the general population as well as matched controls [9, 10, 14, 15]. QoL has been predominantly addressed by validated self-administered questionnaires, such as 36-Item Short Form Health Survey (SF-36), WHO-5 Well-being Index Survey (WHO-5) or Hospital Anxiety and Depression Scale, with evidence of impairment both in physical and mental domains [16–18]. None of these tools are specific for HypoPT and novel disease-specific questionnaires have been recently proposed [19].

However, it is noteworthy that QoL is a general, subjective, multidimensional concept and QoL questionnaires are capable of accessing only the self-perception of an individual lack of well-being as consequence of the disease [20, 21]. A further and more comprehensive approach to the possible cognitive alterations of patients with HypoPT, should be estimated by formal standardized psychometric assessment which evaluates “cognitive functioning”, including attention, language, visual-spatial abilities, and memory. These are specific, trained-personnel administered, standardized tests with population-based data allowing a comparison between individual’s performance and normal range [22]. Neuropsychological tests are a validated diagnostic tool for assessing patients’ cognitive function. They enable clinicians to gain insight into the structural and functional integrity of the brain, to assess the severity of cognitive dysfunction, and to help establish a diagnosis [23]. Unfortunately, very little data are available on HypoPT focusing on the specific cognitive impairment: a recent meta-analysis covered this aspect, but consistent data were available only for idiopathic HypoPT, while for PS-HypoPT only clinical cases were reported [24].

The aims of this study were to evaluate (1) cognitive functions of patients with chronic PS-HypoPT compared to a control population using a standardized neuropsychological approach, and (2) the relationship between cognitive function and biochemical parameters.

## Materials and methods

### Study design and patients

This was an observational, monocentric study with a prospective design. Between March 2018 and July 2020, we evaluated 100 consecutive patients with differentiated thyroid cancer in remission (or without residual disease) who were followed by the routine scheduled monitoring visit at the outpatient clinic of Endocrine Unit of the University Hospital of Pisa. Among them, 40 patients presenting diagnosis of chronic PS-HypoPT and 40 patients without PS-HypoPT (control group), were eligible and asked to participate in the present study. Thirty-three patients with PS-HypoPT and 24 controls accepted and were included in the study. They matched the following inclusion criteria: (1) age > 18 years, (2) willing and able to perform testing (3) absence of residual thyroid disease and optimal control of thyroid hormones under levothyroxine therapy, (4) in PS-HypoPT group: diagnosis since at least 3 years, (5) control group matched for age, sex and educational levels with PS-Hypo group.

Exclusion criteria were (1) known psychiatric disease; (2) assumption of psychoactive drugs; (3) medical history of brain injuries, cerebrovascular or neurological disorders; (4) any condition that interferes with testing; (5) inadequate thyroid hormones control under levothyroxine therapy; (6) pregnancy or lactation.

All patients were asked to undergo biochemical testing and a standardized neuropsychological assessment by a trained psychologist, in the same morning, to ensure that the biochemical measurements and neuropsychological performance reflect the conditions of the patients in that same moment.

The Institutional Review Board approved the study; all patients signed written informed consent.

### Biochemistry

Fasting serum or plasma samples were collected. Serum calcium, creatinine, phosphate, 25-hydroxyvitamin D [25(OH) vitamin D] and plasma PTH were measured as previously described [25, 26]. Serum Alb-Ca) using the following formula [(Alb-Ca = (total serum calcium mg/dl + 0.8 × [4–serum albumin g/dl)] [27]. The estimated glomerular filtration rate was calculated using the EPI-CKD formula [28].

### Neuropsychological assessment

All patients underwent a complete cognitive assessment by performing standardized psychometric tests, each evaluating different cognitive functions: (1) Trail Making Test A, B, B-A for Visual-Spatial attention [29]; (2) Digit Span Forward for Short-Term Verbal Memory [30]; (3) Digit Span Backward for Verbal Working Memory [30]; (4) Block Tapping Test for Short-Term Visual-Spatial Memory [30]; (5) Verbal Fluency Test for phonological skills and (6) Semantic Fluency Test for both Executive Function and Semantic Memory skills [31].

In Table 1, all neuropsychological tests and their significance are summarized.

**Table 1:** Comparison of pathological performances between PSHypo-PT and matched control group

Neuropsychological test	Test significance	Hypoparathyroidism patients (*n* = 33)	Control patients (*n* = 24)	*X*^*2*^	*P *value
Trail-making test-A	Evaluates the selective visual-spatial attentional capacity	ES 0 *n* = 0 (0%) ES 1 *n* = 0 (0%) ES 2 *n* = 1 (3%) ES 3 *n* = 1 (3%) ES 4 *n* = 31 (94%) Total N. = 33	ES 0 *n* = 0 (0%) ES 1 *n* = 0 (0%) ES 2 *n* = 0 (0%) ES 3 *n* = 0 (0%) ES 4 *n* = 24 (100%) Total N. = 24	NA	NA
Trail-making test-B	Evaluates the divided visual-spatial attentional capacity	ES 0 *n* = 0 (%) ES 1 *n* = 0 (%) ES 2 *n* = 0 (%) ES 3 *n* = 4 (12.5%) ES 4 *n* = 28 (87.5%) Total N. = 32	ES 0 *n* = 2 (8.7%) ES 1 *n* = 0 (%) ES 2 *n* = 0 (%) ES 3 *n* = 2 (8.7%) ES 4 *n* = 19 (82.6%) Total N. = 23	2.888	0.089
Trail-making test B–A	Evaluates the capacity of divided visual-spatial attention net of psychomotor component	ES 0 *n* = 0 (0%) ES 1 *n* = 0 (0%) ES 2 *n* = 1 (3.1%) ES 3 *n* = 7 (21.9%) ES 4 *n* = 24 (75%) Total N. = 32	ES 0 *n* = 2 (8.7%) ES 1 *n* = 0 (0%) ES 2 *n* = 1 (4.3%) ES 3 *n* = 3 (13.00%) ES 4 *n* = 17 (73.9%) Total N. = 23	2.888	0.089
Digit span forward	Evaluates the short-Term verbal memory	ES 0 *n* = 1 (3.0%) ES 1 *n* = 0 (%) ES 2 *n* = 3 (9.1%) ES 3 *n* = 11 (33.3%) ES 4 *n* = 18 (54.5%) Total N. = 33	ES 0 *n* = 2 (8.3%) ES 1 *n* = 0 (0%) ES 2 *n* = 5 (20.8%) ES 3 *n* = 5 (20.8%) ES 4 *n* = 12 (50.0%) Total N. = 24	0.784	0.376
Digit span backward	Evaluates the verbal working memory	ES 0 *n* = 1 (3.0%) ES 1 *n* = 1 (3.0%) ES 2 *n* = 6 (18.2%) ES 3 *n* = 9 (27.3%) ES 4 *n *= 16 (48.5%) Total N. = 33	ES 0 *n* = 3 (12.5%) ES 1 *n* = 3 (12.5%) ES 2 *n* = 4 (16.7%) ES 3 *n* = 2 (8.3%) ES 4 *n* = 12 (50.0%) Total N. = 24	1.910	0.167
Block tapping test	Evaluates the visual-spatial short-term memory	ES 0 *n* = 2 (6.5%) ES 1 *n* = 2 (6.5%) ES 2 *n* = 7 (22.6%) ES 3 *n* = 3 (9.7%) ES 4 *n* = 17 (54.8%) Total N. = 31	ES 0 *n* = 2 (8.3%) ES 1 *n* = 4 (16.7%) ES 2 *n* = 5 (20.8%) ES 3 *n* = 2 (8.3%) ES 4 *n* = 11 (45.8%) Total N. = 24	0.071	0.790
Verbal fluency test	Evaluates the skills of phonological lexical access	ES 0 *n* = 0 (0%) ES 1 *n* = 2 (6.1%) ES 2 *n* = 2 (6.1%) ES 3 *n* = 4 (12.1%) ES 4 *n* = 25 (75.8%) Total N. = 33	ES 0 *n* = 0 (0%) ES 1 *n* = 1 (4.3%) ES 2 *n* = 2 (8.7%) ES 3 *n* = 3 (13.0%) ES 4 *n* = 17 (73.9%) Total N. = 23	NA	NA
Semantic fluency test	It is a multi-domain test capable of simultaneously evaluating semantic memory and executive function	ES 0 *n* = 2 (6.1%) ES 1 *n* = 1 (3.0%) ES 2 *n* = 2 (6.1%) ES 3 *n* = 0 (0%) ES 4 *n* = 27 (84.8%) Total N. = 33	ES 0 *n* = 2 (8.3%) ES 1 *n* = 0 (0%) ES 2 *n* = 1 (4.2%) ES 3 n = 0 (0%) ES 4 *n* = 21 (87.5%) Total N. = 24	0.110	0.740

*X*^*2*^ chi square, *ES* equivalent score, *NA* not applicable (no patient, for both groups, reaches an ES = 0), *Total N* total number

Tests were administered to the patients by a trained psychologist (GA): at variance with the questionnaire for QoL, neuropsychological tests need to be administered by a specialist, who is aware of psychometric properties of tests, validity, reliability and to obtain patients’ maximal effort [32]. Patients were evaluated separately (session lasting 45/60 min) and tests were administered in a specific order, using always the same sequence.

Test battery outputs were expressed in Equivalent Scores (ES), representing inferentially inspired scores declined in 5 gradations: 0 = pathological performance, including performance that is in the lower “tail” of the distribution, below the 5th percentile, with a probability of 95% (ie., with a known risk of error of 5%); 1 = borderline area, including performance that falls between the 5th percentile and the 20th percentile.; 2 and 3 = medium–low, including performances that are placed in the central part of the distribution, that is, between the 20th and 50th percentiles.; 4 = normal performance, including performances that are in the upper half of the distribution, i.e., above the median, beyond the 50th percentile. To estimate each ES, raw scores were converted into Weighted Scores (WS) as a function of age and schooling variables [33, 34].

### Statistical analysis

Biochemical variables and neuropsychological test scores were tested for normality using the Shapiro–Wilk tests. The differences of continuous variables between the PS-HypoPT and control group or between PS-HypoPT patients with target Alb-Ca and not at target Alb-Ca (according to guidelines, Alb-Ca = 8.4 mg/dl [35], were evaluated using the *t*-tests or Mann–Whitney test for variable with or without a normal distribution, respectively. The differences of each categorical variable between the PS-HypoPT and control group were evaluated using Chi-square tests. The relationships between WS and biochemical variables in the PS-HypoPT group were assessed using the Pearson or Spearman correlations for variables with or without a normal distribution, respectively. To evaluate the level dependency of biochemical variables, the significant correlations were analysed through one-way Analysis of Variances (ANOVA). In the ANOVA analysis, three groups were compared: control group, PS-HypoPT group with Alb-Ca ≤ 8.9 mg/dl (the threshold was set as the median value) and PS-HypoPT group with Alb-Ca > 8.9 mg/dl. Post-hoc comparisons were used to determine the significant pairs of ANOVAs.

Significant levels were set at the 5% level for statistical analyses; however, regarding post hoc tests, the homogeneity of the variances among the groups was tested (Levene’s test) and each post hoc *p* value was adjusted (Bonferroni correction) for multiple comparisons. Data management and analysis were performed using SPSS (version 24, IBM Software Group) and Matlab (R2017b, Mathworks Inc.).

## Results

### Patients

The PS-HypoPT group included 33 patients, mainly females (23, 69.7%), with a median age of 52 (41–63) years. The median average of the duration of the disease at the time of the evaluation was 6.5 years (4–10). PS-HypoPT patients were treated with conventional therapy: all received calcitriol at a median dosage of 0.75 µg/die (range IQR 0.5–1.0) and 18 (54.5%) also received calcium carbonate at a median dosage of 500 mg/die (range IQR 500–1000). Patients were defined “at target” when the criteria of the recent European Society of Endocrinology guidelines on the management of adult chronic HypoPT were met [35].

The control group included 24 patients without PS-HypoPT, mainly females (*n* = 21, 83.3%) with a median age of 53 (41–58) years and a median duration of the disease 9.5 years (6–13).

Both patients and controls were euthyroid under levothyroxine therapy and well controlled. There was no significant difference in age, gender distribution and education levels between PS-HypoPT patients and controls. Full clinical and biochemical data of patients and controls are reported in Table 2.

**Table 2:** Clinical and biochemical features of patients

Characteristic	Hypoparathyroidism (*n* = 33)	Controls (*n* = 24)	Test		*p value*
Sex					
Female *n* (%)	23 (69.7%)	21 (83.3%)	*X*^2^	2.501	0.114
Males *n* (%)	10 (30.3%)	3 (16.7%)			
Age (years)^a^	52.00 (41.50–63.00)	53.00 (41.25–58.00)	*U*	385.500	0.865
Duration of the disease (years)^a^	6.50 (4.00–10.00)	9.50 (6.25–13.00)	*U*	244.000	0.043*
Education (years)^a^	13.00 (8.00–17.00)	15.00 (13.00–17.00)	*U*	302.000	0.115
Serum_Calcium (mg/dl)^a^	8.85 ± 0.51	9.39 ± 0.31	*t*	− 4.555	0.000*
SAlbCa (mg/dl)^a^	8.9 (8.55–9.15	9.30 (9.10–9.60)	*U*	268.000	0.038*
Ionized calcium	1.15 ± 0.07	1.24 ± 0.03	*t*	− 5.995	0.000*
PTH (pg/ml)^a^	10.00 (7.00–16.00)	29.50 (20.50–36.35)	*U*	50.500	0.000*
25OHD (ng/ml)^a^	33.64 ± 9.18	25.25 ± 8.61	*t*	3.494	0.001*
TSH (µU/ml)^a^	0.45 (0.03–0.96)	0.44 (0.09–1.42)	*U*	338.500	0.353

*U* Mann–Whitney, *t* *t* test, *X*^2^ chi-square; *p value < 0.05

^a^Results are expressed as mean ± SD for parametric values and as median and interquartile range for non-parametric values

### Neuropsychological test performance and correlation with serum calcium

In the PS-HypoPT group, 2 patients (6%) showed a pathological performance (Equivalent Score 0) by at the Semantic Fluency Test, 2 patients (6.5%) at the Block Tapping Test, 1 patient (3%) at Digit Span Forward, and 1 (3%) at Digit Span Backward, but there was not a significant difference with the control group (*p* > 0.05). Overall, the distribution of WS as a function of age and school variables, did not differ between patients and controls across all tests (Table 1).

**Fig. 1:**
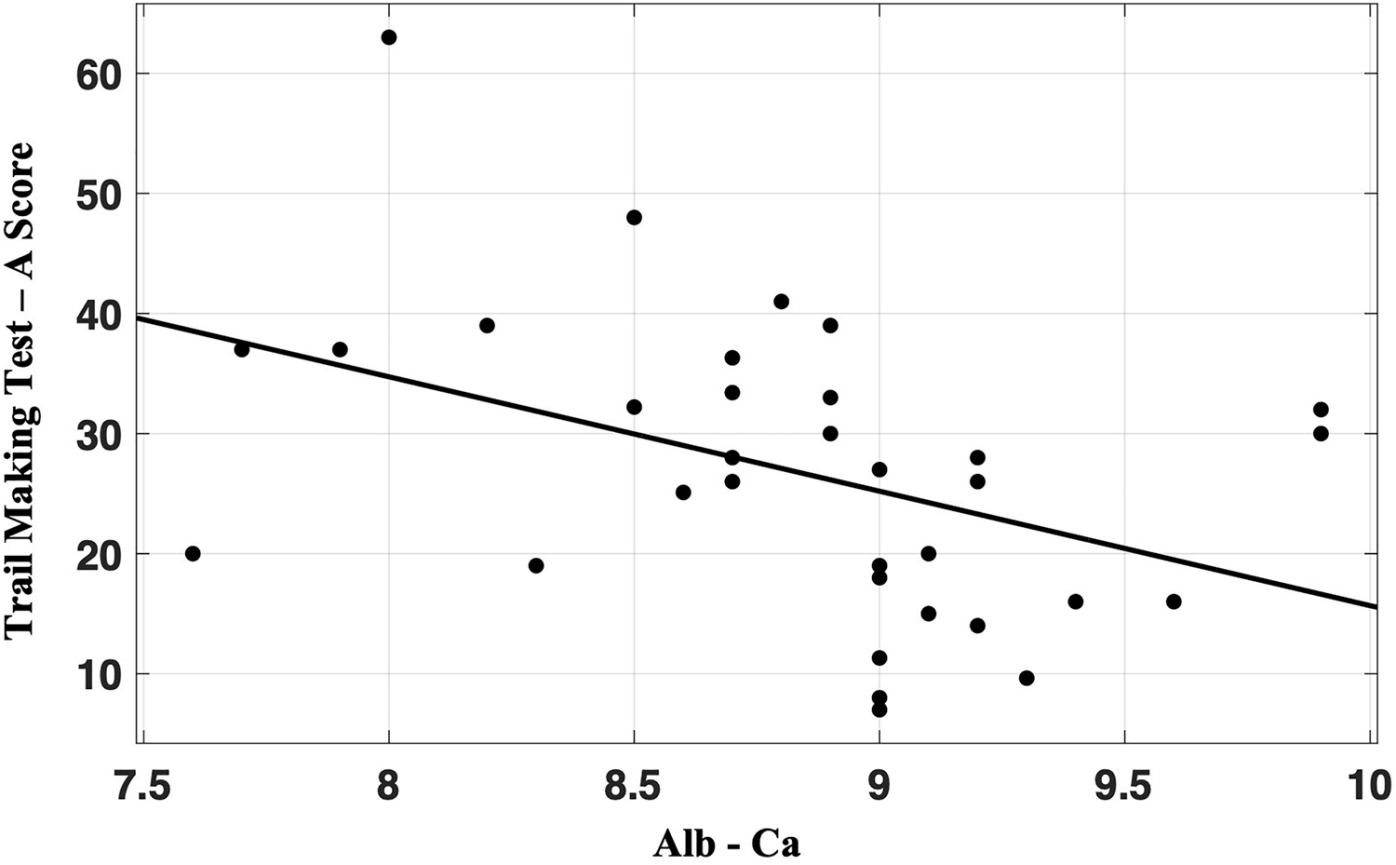
Pearson’s correlation between Trail Making Test-A scores and Alb–Ca levels (*r* = − 0.423; *p* = 0.014) in Post-Surgical hypoparathyroidism patients

**Fig. 2:**
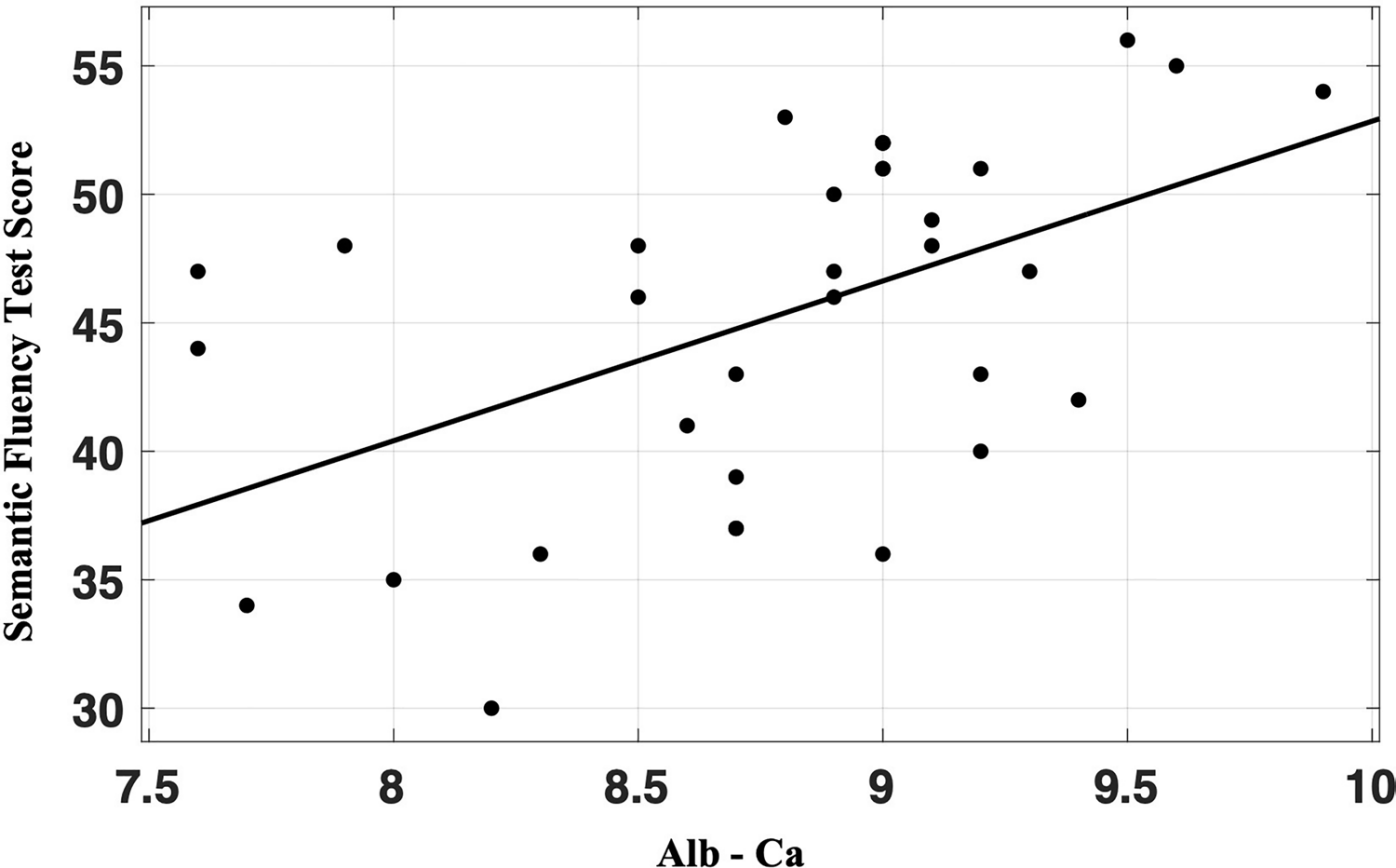
Pearson’s correlation between Semantic Fluency Test scores and Alb-Ca levels (*r* = 0.510; *p* = 0.002) in post-surgical hypoparathyroidism patients

In the PS-HypoPT group, a significant inverse correlation was found between Alb-Ca and Trail Making Test A (TMT-A) scores (*r* = − 0.423; *p* = 0.014). The correlation suggested that the lower the level of Alb-Ca is, the worse is the performance at focused visuo-spatial attention (Fig. 1). Conversely, a positive correlation was present between Alb-Ca and the Semantic Fluency Test scores (*r* = 0.510; *p* = 0.002), suggesting that low serum calcium correlates with worse performance on semantic memory abilities and executive function (Fig. 2). The same correlations were not found in the control group.

**Fig. 3:**
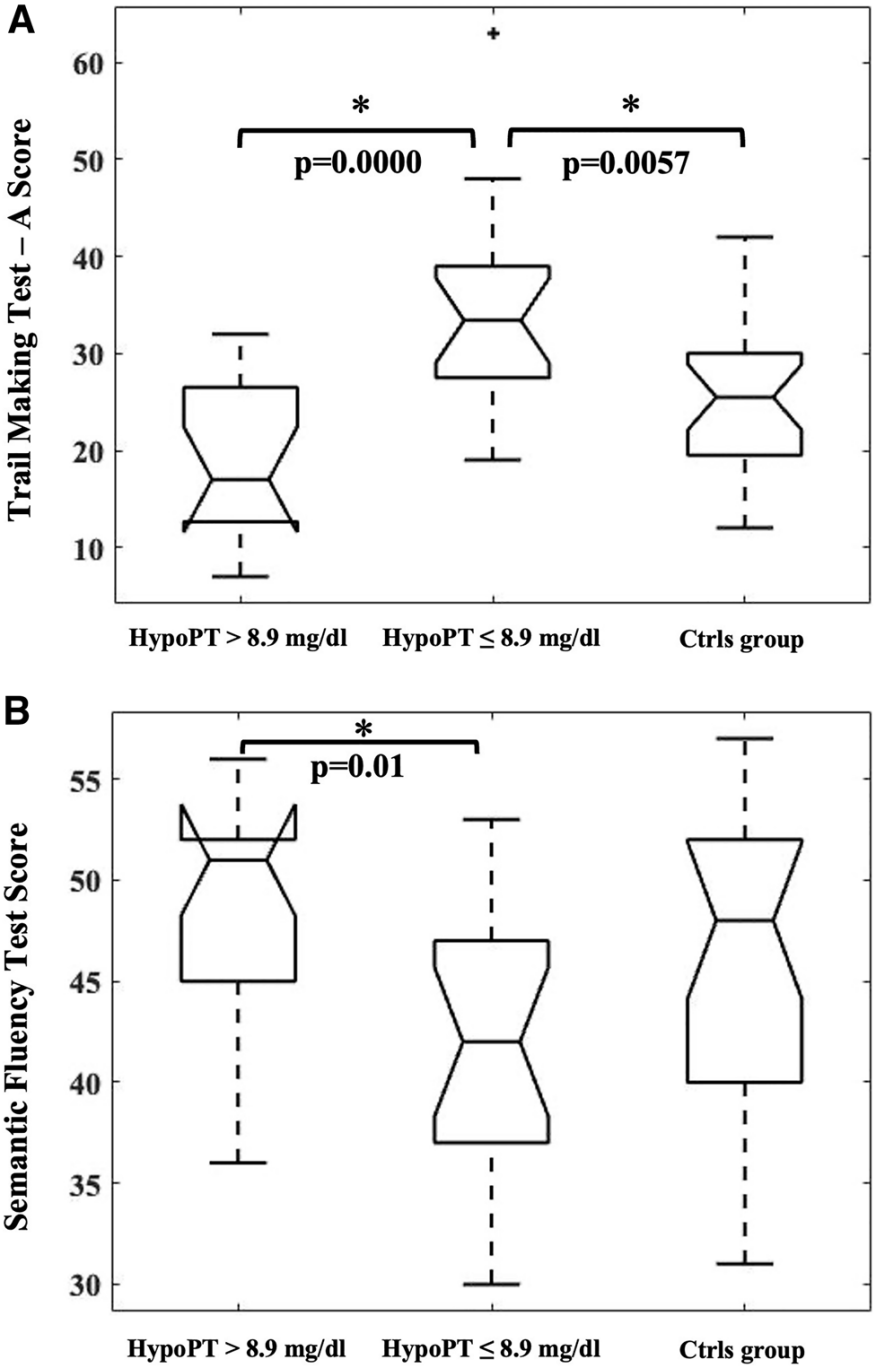
**A** Statistical boxplots of ANOVA tests for focused visuo-spatial attention (Trail Making Test-A), including: post-Surgical hypoparathyroidism patients with Alb-Ca levels > 8.9 mg/dl (PS-HypoPT > 8.9 mg/dl), Post-Surgical hypoparathyroidism patients with Alb-Ca levels ≤ 8.9 mg/dl (PS-HypoPT ≤ 8.9 mg/dl), and control group (Ctrls group). The asterisk with the bar below indicates the boxplots that show a statistically significant difference. **B** Statistical boxplots of ANOVA tests for executive function and semantic memory (Semantic Fluency Test), including post-surgical hypoparathyroidism patients with Alb-Ca levels > 8.9 mg/dl (PS-HypoPT > 8.9 mg/dl), post-Surgical hypoparathyroidism patients with Alb-Ca levels ≤ 8.9 mg/dl (PS-HypoPT ≤ 8.9 mg/dl), and control group (Ctrls group). The asterisk with the bar below indicates the boxplots that show a statistically significant difference

Patients with PS-HypoPT were grouped according to the median Alb-Ca (8.9 mg/dl) (8.55–9.15): 16 patients (48.5%) with values > 8.9 mg/dl and 17 (51.5%) with values ≤ 8.9 mg/dl. These two groups and the control group were compared by ANOVA. Levene’s Test was satisfied (*p* > 0.05). Post-hoc comparisons showed that PS-HypoPT patients with Alb-Ca ≤ 8.9 mg/dl had a significantly lower test performance compared with PS-HypoPT patients with Alb-Ca > 8.9 mg/dl, both at the TMT-A test (higher scores, mean score: 34.53–18.55; *p* < 0.0001, respectively) and Semantic Fluency Test (lower scores, mean score: 41.94–48.68; *p* = 0.01, respectively) (Fig. 3A, B). Moreover, at post hoc analysis, PS-HypoPT patients with Alb-Ca ≤ 8.9 mg/dl had also a significantly lower test performance compared with control patients’ group at TMT-A test for focused visuo-spatial attention (mean score: 34.53–25.5; *p* = 0.0057).

## Discussion

In the recent years, a novel interest into the clinical complications of patients with chronic Hypo-PT treated with conventional therapy has emerged [36, 37]. A high prevalence of renal calcifications (31%) and an increased risk of kidney insufficiency [38–41] have been shown. The skeletal evaluation shows a low bone turnover and impaired microarchitecture, with uncertain fracture risk [3, 8].

Hypo-PT patients also complain about a wide variety of psychological symptoms, including depression, fatigue, anxiety, memory dysfunction [9, 10] depicting a “neuropsychological burden”. One of the most important findings in the literature on the interaction between the aforementioned mental aspects and physical symptoms is the alteration of QoL. In a recent paper by Hamdy et al., more than 70% of patients with Hypo-PT not adequately controlled with conventional therapy showed impaired QoL [42]. Indeed, QoL is a synthetic evaluation of the disease and the effects it has on the patients’ global health [43, 44].

The first aim of this study was to evaluate the changes of cognitive functioning in patients with PS-Hypo-PT compared to control subjects. We investigated Visual-Spatial attention, Short-Term verbal Memory, Verbal Working Memory, Short-Term Visual-Spatial Memory, phonological skills, Executive Function, Semantic Memory skills and no statistical differences were detected comparing to control patients.

For better characterizing the role of the alteration of calcium homeostasis in favouring the “neuropsychological burden” in our cohort of PS-Hypo-PT, we studied how Alb-Ca might affect different cognitive functions. In this study, we could observe that serum calcium levels are associated with selective attention skills: as serum calcium levels increase (mantaining the normal range), graphomotor speed and visual scanning capacity increases in the attentional task [45]. Moreover, we could observe that serum calcium levels are also associated with the simultaneously executive and semantic integrative function [46] as serum calcium levels increases, patients maintain a good capacity for working memory, inhibitory control, set shifting and access to semantic store.

At the best of our knowledge, this is the first study evaluating, in a such rigorous methodological approach, some cognitive domains in the clinical model of chronic PS-HypoPT. In a very recent systematic review [24], mainly describing patients with idiopathic HypoPT, a relationship between HypoPT and cognitive impairment was demonstrated. The cross-sectional study by Aggarwal et al. also showed a greater alteration of cognitive functioning in 62 patients with idiopathic HypoPT, younger than our patients (mean age 24.5 ± 4.1, females 43.5%) who were followed for 12.0 ± 8.7 years, and 70 controls. In this study, the alterations of cognitive functions were greater than in our cohort of patients with PSHypoPT. Moreover, in the cohort of Aggarwal et al., cognitive dysfunctions were detected in one third of patients [47], whereas in our cohort, patients rarely reached a pathological score in  one test and there was no difference with control patients. The different etiopathogenesis of HypoPT (idiopathic vs post-surgical) and the greater duration of the disease (12 years vs 6 years) could account for such differences.

Calcium plays a central role in the modulation of fundamental neurophysiological processes [48]. It regulates neural-cell homeostatic functions: membrane excitability [49], dendrite development [50], synaptogenesis [51]cell proliferation and cell death [52]. All synaptic phenomena, from neurotransmitter release to Long-Term Potentiation (LTP), are heavily related to calcium modulation exerted by neurons and astrocytes [53]. Intracellular calcium waves, for example, allow the astrocytes to create a network able to support integrative brain functions on a large scale, from dynamic glucose delivery to cognitive information processing [54]. From a cognitive standpoint, alteration in calcium homeostasis is involved in pathophysiology of cognitive decline and dementia [55, 56]. Since serum Alb-Ca correlates with serum ionized calcium [57] and Ca influx to the CSF and brain seems to be linearly related to the serum ionized calcium concentration [58] we assume that low serum calcium levels, due to the lack of PTH, might jeopardize short and large-scale integration. This could be the pathophysiological mechanism underlying the alteration of cognitive functions and their related networks in untreated patients with PS Hypo-PT; the same scenario could be active in our patients, but the normal serum calcium in our patients would prevent the occurrence of detectable pathological abnormalities.

A potential direct role of PTH on the central nervous system and brain has been proposed as possibly pivotal in the etiopathogenesis of neurocognitive symptoms in patients with HypoPT [24]. The PTH family of peptides includes the products of 3 genes: PTH, parathyroid hormone related protein (PTHrP), and the PTH receptor 2 (PTHR2) agonist tuberoinfundibular peptide 39 (TIP39). This is a group of structurally similar polypeptides, involved in bone homeostasis but also many other functions in the mammalian body [59]. They use different receptors: the PTHR1 mediates the classical effects of PTH on bone and kidney, while the PTH2R, which is expressed in central nervous system areas crucial for cognitive development and maintenance [60], such as hypothalamus, amygdala, pontine tegmentum and thalamic nuclei, has been proposed to be also involved in sensory, limbic and neuroendocrine functions [61, 62]. Interestingly, PTH is not the only ligand for PTHR2. The TIP39 activates the PTHR2 even more potently than PTH (or PTHrP) and animal studies suggest its involvement in modulation of emotional process and memory [63, 64]. This observation raises the possibility that the neurocognitive alterations in patients with chronic HypoPT could be mediated, at least in part, by the lack of interaction of PTH (or PTHrP) with the PTHR2.

Ultimately, the emerging role of PTH in the brain has pushed research to deeply investigate the mechanisms of neuropsychological involvement during Hypo-PT and sophisticated approaches using magnetic resonance imaging (MRI) in clinical trials which are still ongoing. In this context, the aim of this study was to evaluate cognitive alterations of patients with PS-HypoPT using a neuropsychological standardized approach. This allows to reveal more refined alterations of cognitive functions in patients with HypoPT and their relationship with biochemical parameters.

The strength of this study are as follows: (1) this is the first study that evaluates cognitive impairment in patients with chronic PS-HypoPT using a psychometric approach with specific clinical psychologist-trained tests battery, (2) the presence of a control population, (3) correlation between performance at psychological tests with biochemical parameters.

This study has also some limitations, namely the cohort is relatively small, and it is a cross-sectional study, so we have no follow-up data with possible improvement of cognitive performance with improvement of biochemical parameters. Moreover, only one serum calcium measurement performed at the same moment of the neuropsychological testing is available. However, we can state that serum calcium in patients with Hypoparathyroidism in this study is quite stable since the difference between calcium measured at neuropsychological evaluation and calcium measured within the previous year without substantial therapy changes, was not significant (*p* = 0.09).

In conclusion, we show that patients with chronic PS-HypoPT treated with conventional therapy do not show a severe cognitive impairment, which is frequent in patients with idiopathic HypoPT. Nonetheless, some cognitive functions such as visuo-spatial attention, executive function and semantic memory appear to be modulated by Alb-Ca and endangered by its low levels. In addition to the possibility to extend this psychometric approach to the clinical evaluation of patients with PS-HypoPT, this research could be helpful to focus on slight refined cognitive alterations in chronic PS-HypoPT, which could be better investigated using more complex tools, such as MRI.

## Data Availability

The datasets generated during and/or analysed during the current study are available from the corresponding author on reasonable request.
